# The Changes of the Endophytic Bacterial Community from Pepper Varieties with Different Capsaicinoids

**DOI:** 10.3390/microorganisms13030596

**Published:** 2025-03-05

**Authors:** Yuxiang Peng, Xiulan He, Yu Tao, Chi Zhou, Xin Li

**Affiliations:** 1 Longping Branch, College of Biology, Hunan University, Changsha 410125, China; pyxxxx0730@163.com (Y.P.); hexlannnnn@126.com (X.H.); 2 Hunan Institute of Microbiology, Changsha 410009, China; ty123@webmail.hzau.edu.cn; 3 Hunan Engineering Research Center on Excavation and Utilization of the Endophytic Microbial Resources of Plants, Changsha 410125, China

**Keywords:** pepper, varieties, capsaicinoids, endophytic bacteria, community

## Abstract

Capsaicinoids, the key compounds responsible for pepper pungency, have significant commercial and health value, yet the role of endophytic bacteria in their biosynthesis remains unclear. This study investigated the relationship between endophytic bacterial communities and capsaicinoid content across 100 Capsicum annuum varieties. Two high-capsaicinoid (35.0 and 24.8 mg/g) and two low-capsaicinoid (0.8 and 0.9 mg/g) varieties were selected for 16S rRNA sequencing and microbial analysis. High-capsaicinoid varieties exhibited greater bacterial richness and diversity compared to low-capsaicinoid varieties. Taxonomic profiling revealed distinct community compositions: *Enterobacter*, *Bacteroides*, and *Escherichia_Shigella* were enriched in high-capsaicinoid fruits and positively correlated with capsaicinoid levels, while *Chujaibacter* and *Brochothrix* dominated the low-capsaicinoid varieties. Functional annotation highlighted nitrogen-fixing bacteria as more abundant in high-capsaicinoid varieties. Inoculating peppers with isolated *Enterobacter* strains significantly increased capsaicinoid content, confirming its role in biosynthesis. These findings demonstrate that the pepper genotype shapes endophytic bacterial communities, which in turn influence capsaicinoid production through metabolic- and nitrogen-associated pathways. This study provides foundational insights into microbiome-mediated enhancement of pepper pungency, offering potential strategies for agricultural and industrial applications.

## 1. Introduction

Capsicum species are among the most popular vegetables and spices worldwide owing to their significant economic and nutritional value. The Capsicum genus consists of over 30 species, with three widely cultivated varieties known for their pungent fruits: *Capsicum annuum*, *Capsicum chinense*, and *Capsicum frutescens* [1,2]. The pungency of these fruits is mainly attributed to the accumulation of capsaicinoids, with capsaicin and dihydrocapsaicin being the major components, accounting for nearly 90% of the total capsaicinoids [3]. Capsaicinoids have a variety of functions, including analgesic, anti-cancer, anti-inflammatory, antioxidant, and anti-obesity characteristics [4,5,6]. Additionally, capsaicinoids exhibit anti-fungal and anti-oomycete activities, especially against Fusarium [7]. Thus, increasing the capsaicinoid content in peppers is of great importance.

Capsaicinoid levels vary dynamically during fruit development and are influenced by the genotype or cultivar [8]. Capsicum cultivars exhibit significant variation in pungency. A study of 12 varieties from three species (*C. annuum*, *C. chinense*, and *C. frutescens*) revealed substantial differences in capsaicinoid content [9]. Moreover, the pungency of pepper fruits is also affected by environmental factors, including abiotic factors such as soil type, osmotic properties, nutrient composition, light, temperature, and water, as well as biotic factors like microorganisms [9,10].

Endophytes are microorganisms that colonize the tissues and organs of healthy plants at certain or all stages of their life cycles without causing significant damage to the host plants [11]. Endophytes provide various benefits to host plants, such as promoting growth and health, enhancing nutrient uptake, improving tolerance to abiotic stresses, and protecting against pathogens [12,13,14]. Previous studies have reported that plant genotypes, organs (e.g., roots, leaves, and fruits), growth stages, and geographical locations have a substantial impact on the formation of microbial diversity and community structures in plants [15,16,17,18,19]. However, research on how different pepper varieties affect the structure and functions of endophytic microorganisms remains limited.

Microorganisms play a crucial role in modulating plant secondary metabolism [20]. Plant growth-promoting rhizobacteria (PGPR) have been shown to significantly increase the accumulation of proline in soybean roots [21] and flavonoids and kinsenoside in *Anoectochilus roxburghii* [22]. In addition to rhizosphere bacteria, numerous studies have reported that endophytic bacteria also regulate the synthesis of plant secondary metabolites, including terpenoids, phenols, and alkaloids [23]. For example, the inoculation of medicinal plants with endophytic bacteria such as *Acinetobacter*, *Bacillus*, and *Pseudomonas* can promote the synthesis of terpenoids [24,25,26,27]. *Achromobacter*, an endophytic bacterium of *Polygonum cuspidatum*, enhances the accumulation of polydatin within roots [28]. Alkaloids are nitrogen-containing heterocyclic compounds primarily derived from amino acids [29]. Several studies have revealed that endophytic bacteria influence plant alkaloid metabolism. For instance, the endophytic bacteria Acinetobacter and Marmoricola are involved in regulating the biosynthesis of phenylisoquinoline alkaloids (BIAs) in poppy [30], and the co-inoculation of these two bacteria significantly increased the morphine content in poppy. The authors of [31,32] demonstrated that *Micrococcus*, an endophytic bacterium isolated from *Catharanthus roseus*, significantly increased the contents of key terpenoid indole alkaloids like vindoline in the leaves and ajmalicine in the roots. Capsaicinoids, a type of alkaloid synthesized in the placenta of pepper fruits, have not yet been reported to be influenced by plant endophytic bacteria in terms of their biosynthesis.

In the study, we used 16S rRNA gene sequencing to analyze fruit-associated endophytic bacterial communities in four pepper varieties exhibiting varying pungency levels. The objectives of this research were threefold: (i) to characterize the structure and composition of endophytic bacterial communities within these pepper varieties, correlating them with differing capsaicinoid contents; (ii) to investigate the potential influence of endophytic microorganisms on capsaicinoid biosynthesis; and (iii) to identify specific beneficial microorganisms capable of enhancing capsaicinoid production. This study will help us understand how pepper genotypes and characteristics impact the interaction between endophytic microbes with host plants.

## 2. Materials and Methods

### 2.1. Plants and Endophytic Bacteria Materials

One hundred pepper varieties, all belonging to *C. annuum*, were obtained from the Hunan Vegetable Research Institute (HVRI). These peppers were planted at the Gaoqiao experimental field of HVRI (N 28°35′, E 113°14′). Pepper fruit samples were obtained during the green-ripening phase. Three biological replicates were conducted for each pepper variety. Each sample was divided into three portions: one for capsaicinoid content determination (stored at 4 °C), one for sequencing analysis (stored at −80 °C), and one for endophyte isolation (stored at −80 °C).

### 2.2. Isolation and Identification of Endophytes from the Fruits of Peppers

Endophytes were isolated from pepper fruits. The pepper fruits were subjected to surface sterilization using a 4% NaClO solution for 5 min, followed by five rinses with sterile distilled water. Subsequently, the fruits were then crushed in a sterile mortar and pestle with 5 mL of a 0.85% sterile saline solution and serially diluted to 10^−6^. A 100 μL aliquot from each dilution was spread on Luria–Bertani agar plates, followed by incubation at 28 °C for 24–72 h. The isolated endophytes were identified via 16S rRNA sequencing at Bioengineering (Shanghai) Co., Ltd. (Shanghai, China). Sequence analysis was performed using nucleotide BLAST (NCBI) (https://blast.ncbi.nlm.nih.gov/Blast.cgi, accessed on 12 July 2024). The strains were stored in a 25% glycerol solution at −70 °C until use.

### 2.3. Pot Experiments and Bacterial Inoculation

Bacteria were cultured in Luria–Bertani broth at 30 °C with agitation (220 rpm) for 24 h. The cells were collected and resuspended in sterile double-distilled water, and the concentration was adjusted to 1.0 × 10^8^ CFU/mL. Surface-sterilized pepper seeds were initially sown in a nutrient substrate. Subsequently, at the eight-leaf stage, seedlings were transplanted into pots (15 cm diameter × 15 cm high) filled with sterilized nutrient soil. The soil medium comprised a 2:1:1 (*v*/*v*/*v*) mixture of topsoil, vermiculite, and perlite. Each pot was irrigated with 20 mL of the bacteria suspension or the same amount of water. Subsequently, 10 mL of the bacteria suspension was applied to the roots, and the same amount was sprayed on the leaves every five days for three times. During the flowering stage, 10 mL of the bacteria suspension was sprayed on the peppers every two days. Other peppers were sprayed with the same amount of sterile double-distilled water and served as the control. All inoculated and control peppers were randomly arranged in the growth chamber. The growth chamber conditions are set to 25 °C with a 12 h photoperiod, 60% relative humidity, and an average midday photosynthetic active radiation of 1000 μmol m^−2^ s^−1^. Each treatment had six biological replicates. At the mature green stage, fruits were collected for capsaicinoid content determination.

### 2.4. Determination of Capsaicinoid Content

To analyze capsaicinoid content, placental tissue samples were first dried and ground into a fine powder. A 0.1 g aliquot of the powdered tissue was then extracted with 20 mL of acetonitrile using ultrasonic assistance at 65 °C for 20 min. Following extraction, the mixture was centrifuged at low speed, and the resulting supernatant was filtered through a 0.2 μm filter. High-performance liquid chromatography (HPLC, LC-20AT, Shimadzu, Kyoto, Japan) was employed for capsaicinoid quantification. Specifically, capsaicin standards and extracted samples were injected (10 μL) into a Shim-pack GIST C18 column (250 × 4.6 mm, 5 μm particle size) maintained at 30 °C. An isocratic mobile phase consisting of water and acetonitrile was used at a flow rate of 1.0 mL/min over a 30 min run period. Capsaicinoids were detected using a UV detector (Perkin Elmer, Shanghai, China) set to a wavelength of 222 nm [33].

### 2.5. DNA Extraction, Amplification, and Endophyte Sequencing

Before DNA extraction, the samples underwent surface sterilization using a 70% ethanol solution for 30 s, followed by three rinses with sterile, deionized water. All disinfected samples were immediately placed on ice and then stored at −80 °C until total DNA extraction. Three replicates were conducted for each pepper variety. Total genomic DNA was extracted using the TGuide S96 Magnetic Soil/Stool DNA Kit (Tiangen Biotech (Beijing) Co., Ltd., Beijing, China) according to the manufacturer’s instructions. The extracted DNA’s quality and quantity were evaluated using electrophoresis on a 1.8% agarose gel. Additionally, DNA concentration and purity were determined using a NanoDrop 2000 UV-Vis spectrophotometer (Thermo Scientific, Waltham, MA, USA). The bacterial 16S V3-V4 region was amplified using the primer pair 341F/805R (5′-CCTACGGGNGGCWGCAG-3′/5′-GACTACHVGGGTATCTAATCC-3′). The amplified products were purified using the Omega DNA purification kit (Omega Inc., Norcross, GA, USA) and quantified using Qsep—400 (BiOptic, Inc., New Taipei City, Taiwan, ROC). The amplicon library was paired-end sequenced (2 × 250) using an Illumina novaseq6000 system (Beijing Biomarker Technologies Co., Ltd., Beijing, China). The sequence data were deposited in the NCBI Sequence Read Archive (SRA) database.

### 2.6. Data Processing and Statistical Analysis

Bioinformatics analyses for this study were conducted using BMKCloud (http://www.biocloud.net/, accessed on 22 August 2024). Initially, raw sequence data were filtered for quality based on single nucleotide scores using Trimmomatic (v.0.3) [34]. Subsequently, primer sequences were identified and removed with Cutadapt (v.1.9.1) [35]. The resulting paired-end (PE) reads were then assembled using USEARCH (v.10) [36], followed by the removal of chimeric sequences using UCHIME (v.8.1) [37]. High-quality reads, generated from these preceding steps, were then clustered into operational taxonomic units (OTUs) based on a sequence similarity threshold of 97% using USEARCH (v.10). Finally, OTUs with an abundance of less than two counts across all samples were discarded. The analysis of alpha and beta diversities was conducted using QIIME2 [38]. Visualizations were created with the R software (v.3.5.2) [39]. Beta diversity was assessed using unweighted UniFrac distances [40] and visualized through principal coordinate analysis (PCoA) [41]. To assess the statistical significance of observed differences between groups, an analysis of similarities (ANOSIM) was conducted using the Vegan package (v.2.3.0) in the R software (v. 3.4.3). Specifically, ANOSIM R and *p* values were determined through a permutation test with 999 iterations. Furthermore, to identify taxa that significantly differentiated the groups, linear discriminant analysis effect size (LEfSe) [42] was employed. A logarithmic LDA score threshold of 4.0 was used to define discriminative features. To explore the dissimilarities of the microbiome among different factors, a redundancy analysis (RDA) was performed in R using the package ‘vegan’. To identify significant differences in species abundances between groups, Welch’s *t*-test with Benjamini–Hochberg FDR correction was applied using STAMP v.2.1.3 [43]. The correlation network analysis of microbes and capsaicin content was conducted using Cytoscape v.3.7.2 based on Pearson’s correlation analysis (|r|> 0.6, *p* < 0.05). Functional predictions of the microbial flora were performed using the FAPROTAX software (v. 1.1) [44]. The means and standard deviations were calculated utilizing SPSS v.19.0. Statistical significance was computed using a two-tailed Student’s *t*-test. The significance cutoff was as follows: * *p* < 0.05, ** *p* < 0.01, and *** *p* < 0.001. Figures were created using GraphPad Prism v. 9.5.

## 3. Results

### 3.1. Capsaicin Content of Different Pepper Varieties

In this study, four pepper varieties (YMH80, HP03, TJ18, and TJ43) were selected for analysis. The capsaicin (CAP) content in the fruits of 32 varieties was initially assessed (Supplementary Figure S1). Subsequent quantification revealed significant variation in CAP among the selected varieties. Specifically, YMH80 and HP03 exhibited substantially higher capsaicin concentrations (35 mg/g and 24.8 mg/g, respectively) compared to TJ18 and TJ43 (0.8 mg/g and 0.9 mg/g, respectively).

### 3.2. Diversity of Microbial Community

**Figure 1:**
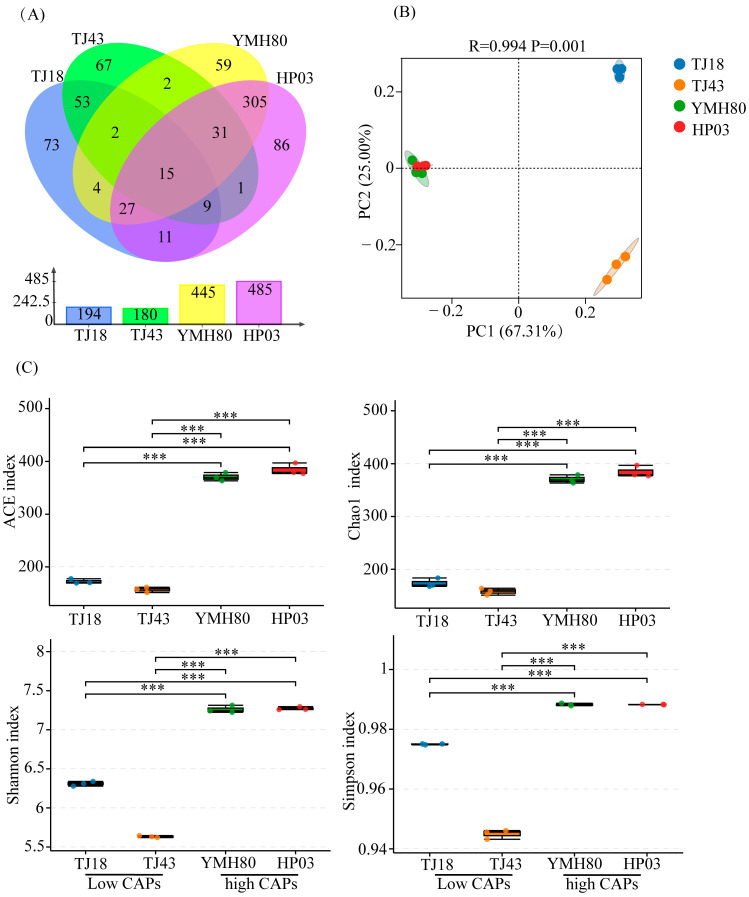
The diversity evaluation of endophytic bacterial communities in four pepper varieties. (**A**) Venn diagrams illustrating the number of endophytic bacterial OTUs in four pepper varieties; (**B**) PCoA for four pepper varieties; (**C**) Alpha diversity indices of endophytic bacteria in four pepper varieties (Tukey’s test revealed significant differences, *** *p* < 0.001).

After quality control and chimera sequence removal, a total of 447,813 bacterial sequences were obtained. These high-quality sequences were clustered into 745 bacterial operational taxonomic units (OTUs). There were 445, 485, 194, and 180 OTUs in YMH80, HP03, TJ18, and TJ43, respectively. It was worth noting that the unique OTUs in high-CAP varieties (YMH80 and HP03/450 OTUs) were more than that in low-CAP varieties (TJ18 and TJ43/193 OTUs) (Figure 1A). Alpha diversity metrics, including the Chao1, ACE, Shannon, and Simpson indices, were calculated to assess the richness and diversity of endophytic bacterial communities. Specifically, Chao1 and ACE values were used as indicators of sample richness, while the Shannon and Simpson indices reflected sample diversity. The results revealed a significantly greater richness and diversity of endophytic bacteria in the high-CAP varieties, YMH80 and HP03, compared to the low-CAP varieties, TJ18 and TJ43 (Figure 1C). This observation was further supported by rarefaction curves and rank abundance curves, which demonstrated significant differences in the richness and diversity of endophytic bacterial communities between the high CAP and low-CAP varieties (Supplementary Figure S2). Principal coordinate analysis (PCoA) revealed significant differences in endophytic bacterial community composition among plant varieties (ANOSIM: R = 0.994, *p* < 0.05; Figure 1B). The first two components (PC1 and PC2) accounted for 67.31% and 25% of the total variance, respectively (Figure 1B). The endophytic bacterial community in the high-CAP varieties was completely separated from those in the low-CAP varieties.

### 3.3. Microbial Community Composition

**Figure 2:**
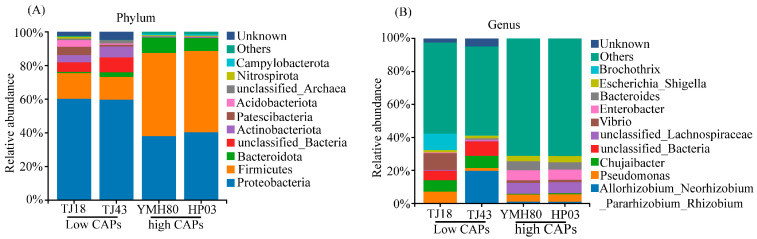
Taxonomic composition of endophytic bacterial communities of four pepper varieties: (**A**) the phylum level and (**B**) the genus level.

**Figure 3:**
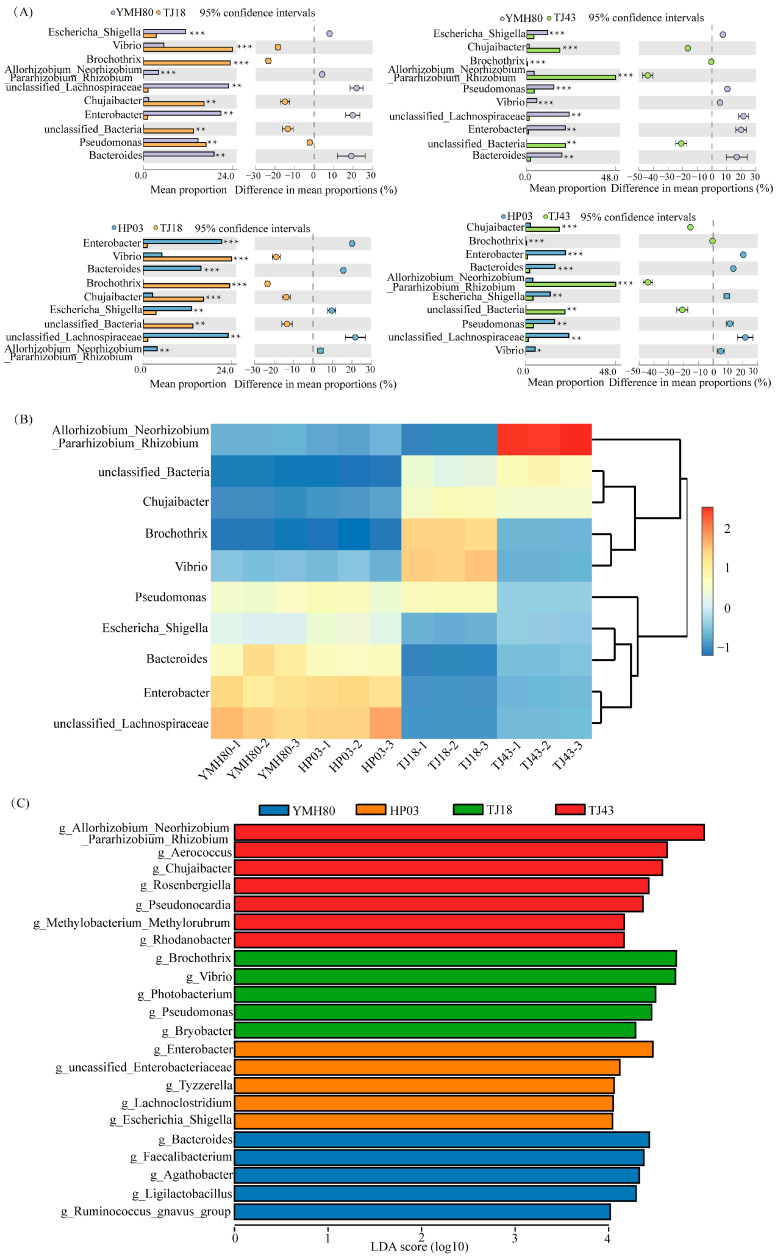
Taxonomic assignments and percent of community abundance at the genus level in the endophytic bacteria of different pepper varieties. (**A**) Comparison of genera exhibiting significant differences among different pepper varieties. The data were visualized by using STAMP (error bars represent Welch’s t-interval, with * *p* < 0.05, ** *p* < 0.01, and *** *p* < 0.001); (**B**) heatmap showing the abundance profile of dominant bacteria (top 10 genera); (**C**) bacterial genus biomarkers in pepper endophytes. The Kruskal–Wallis rank sum test identified species with significant differences between groups at an alpha level of 0.05 and a threshold of 3.5.

At the phylum level, the endophytic bacteria of four different pepper varieties were mainly *Proteobacteria*, *Firmicutes*, *Bacteroidota*, and *Actinobacteriota*, accounting for more than 80% of the OTUs (Figure 2A). *Proteobacteria* comprised the largest proportion of OTUs, ranging from 38.11% to 60.33%. At the genus level, the endophytic bacteria were mainly composed of *Allorhizobium_Neorhizobium_Pararhizobium_Rhizobium*, *Pseudomonas*, *Chujaibacter, unclassified_Lachnospiraceae*, *Enterobacter*, *Bacteroides*, and *Escherichia_Shigella* in all varieties (Figure 2B). The heat map analysis revealed distinct endophytic bacterial communities in high-CAP varieties (YMH80 and HP03) compared to low-CAP varieties (TJ18 and TJ43). At the phylum level, the high-CAP varieties mainly clustered *Firmicutes* and *Bacteroidota*, while the low-CAP varieties mainly clustered *Proteobacteria* and *Actinobacteriota* (Supplementary Figure S3A). At the genus level, the high-CAP varieties mainly clustered *Pseudomonas*, *Enterobacter*, *Bacteroides*, and *Escherichia_Shigella*, while the low-CAP varieties mainly clustered *Chujaibacter* and *Brochothrix* (Figure 3B). Furthermore, *t*-tests were performed to compare the bacterial composition of high-CAP varieties (YMH80 and HP03) with that of low-CAP varieties (TJ18 and TJ43). The analysis revealed significant differences in the relative abundance of several phyla. Specifically, *Firmicutes, Bacteroidota*, and *Campylobacterota* were significantly more abundant in the high-CAP varieties compared to the low-CAP varieties. Conversely, *Proteobacteria, Actinobacteriota, Patescibacteria,* and *Acidobacteriota* were significantly less abundant in the high-CAP varieties. (*p* < 0.001, Supplementary Figure S3B). *Enterobacter*, *Bacteroides*, and *Escherichia_Shigella* were found to be more abundant in the high-CAP varieties compared to the low-CAP varieties. On the contrary, *Chujaibacter* and *Brochothrix* were more prevalent in the low-CAP varieties (*p* < 0.001, Figure 3A). Moreover, *Pseudomonas* exhibited significantly higher relative abundance in YMH80 and HP03 than in TJ43 (*p* < 0.001, Figure 3A). To further identify endophytic bacteria with significant differences among varieties, LEfSe analysis based on LDA was conducted at both the phylum and genus levels. At the phylum level, Firmicutes emerged as a key biomarker in YMH80. While *Patescibacteria* and *Proteobacteria* were the main biomarkers in TJ18 and TJ43 (Supplementary Figure S3C). At the genus level, *Bacteroides* and *Enterobacter* were the main bacterial biomarkers in YMH80 and HP03, respectively. *Brochothrix* and *Allorhizobium_Neorhizobium_Pararhizobium_Rhizobium* were the main bacterial biomarkers in TJ18 and TJ43, respectively (Figure 3C).

### 3.4. Relationships Between Capsaicinoid Content and Microbial Communities

**Figure 4:**
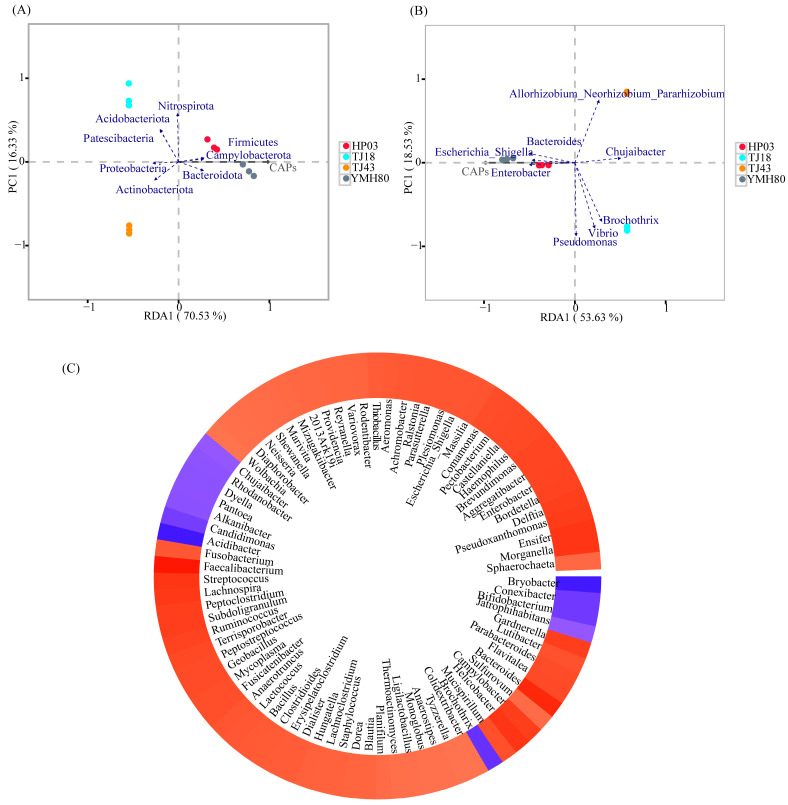
Relationships between capsaicinoid content and endophytic bacterial communities. (**A**) Redundancy analysis of endophytic bacterial communities based on relative abundance at the phylum level and capsaicin content in individual samples; (**B**) redundancy analysis of bacterial communities based on relative abundance at the genus level and capsaicin content in individual samples; (**C**) heatmap of the correlation between capsaicin content and endophytic bacterial genus.

The correlation between CAP and endophytic bacterial composition was analyzed using RDA (Figure 4). At the phylum level, CAP was positively correlated with the relative abundances of *Firmicutes*, *Bacteroidota*, and *Campylobacterota*. However, CAP was negatively correlated with the relative abundances of *Proteobacteria* and *Actinobacteriota* (Figure 4A). At the genus level, CAP showed a positive correlation with *Enterobacter*, *Bacteroides*, and *Escherichia_Shigella*, whereas CAP was negatively correlated with *Chujaibacter*, *Allorhizobium_Neorhizobium_Pararhizobium_Rhizobium*, and *Brochothrix* (Figure 4B).

Furthermore, the co-occurrence patterns of CAP and microbial genera were explored based on strong (Spearman |r| > 0.6) and significant (*p* < 0.01) relationships. A total of 83 genera were significantly affected by CAP (Figure 4C). Notably, the majority of these genera belong to the dominant phyla *Proteobacteria, Firmicutes*, *Actinobacteria*, *Bacteroidota*, and *Campylobacterota*. Among the top ten genus levels, CAP was positively associated with *Escherichia_Shigella* (0.818), *Enterobacter* (0.874), and *Bacteroides* (0.944) and was negatively associated with *Chujaibacter* (−0.727).

### 3.5. Functional Annotation of Bacterial Community Based on the OTU Level

**Figure 5:**
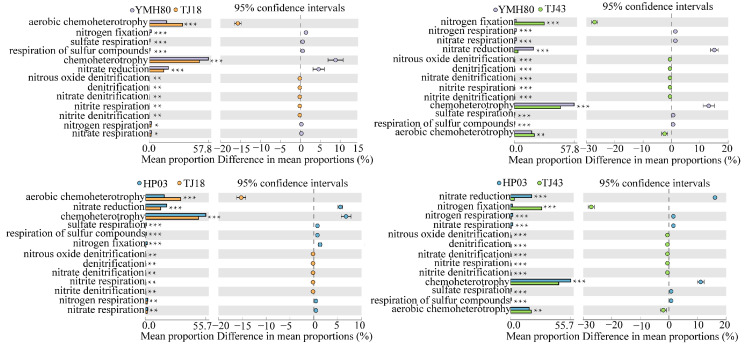
Functional predictions of endophytic bacteria in different pepper varieties (Welch’s *t*-test, * *p* < 0.05, ** *p* < 0.01, *** *p* < 0.001).

We used the FAPROTAXS software to investigate the functional annotation of endophytic bacterial communities for different CAP varieties (Figure 5). The highest abundance of bacteria was related to chemoheterotrophy, aerobic-chemoheterotrophy, nitrate_reduction and nitrogen_fixation, indicating that the microorganisms were closely related to nitrogen metabolism. Microorganisms related to the aerobic-chemoheterotrophy, denitrification, nitrate denitrification, nitrite_denitrification, nitrite_respiration, nitrous_oxide_denitrification categories were significantly increased, whereas those related to chemoheterotrophy, sulfate_respiration and respiration_of_sulfur_compounds were significantly reduced in low-CAP varieties (TJ18 and TJ43) compared to those of the high-CAP varieties (YMH80 and HP03). YMH80 and HP03 exhibited a higher abundance of nitrogen-fixation bacteria but a lower abundance of bacteria related to nitrate reduction, nitrate respiration, and nitrogen respiration compared to TJ18. In conclusion, our results indicate significant differences in the functions of endophytic bacterial communities between low-CAP varieties (TJ18 and TJ43) and high-CAP varieties (YMH80 and HP03).

### 3.6. Endophytic Bacteria Enterobacter Improved the CAP Level of Peppers

**Figure 6:**
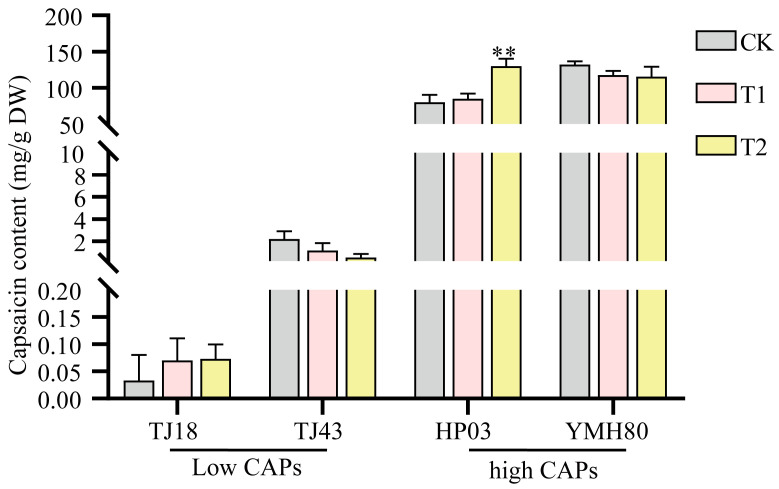
Influences of *Enterobacter* on the capsaicin level in peppers. The error bars indicate the standard deviation (** *p* < 0.01, Student’s *t*-test).

Some isolated endophytes were identified via 16S rRNA gene sequencing. Among them, we isolated a top10 endophytic bacterium, *Enterobacter*, which was significantly more abundant in the high-CAP varieties than in the low-CAP varieties. To test whether endophytic bacteria affect capsaicin content, peppers sprayed with the bacterial suspension were used to test the influences of *Enterobacter* on plant capsaicin content. As shown in Figure 6, inoculation with *Enterobacter* significantly increased the capsaicin accumulation compared to the control in the high-CAP varieties. However, the impact of *Enterobacter* was not statistically different among low-CAP varieties. These results indicated that *Enterobacter* promoted capsaicin synthesis in peppers.

## 4. Discussion

Endophytic bacteria represent vital microbial resources, possessing numerous characteristics crucial for promoting host plant growth [45]. In this study, the number of operational taxonomic units (OTUs) of endophytic bacteria in high-capsaicinoid (CAP) pepper varieties (930) was significantly greater than that in low-CAP pepper varieties (374) (Figure 1A). This preliminary finding reveals the differences in endophytic bacteria between high- and low-CAP varieties at the OTU level. The alpha diversity indices (ACE, Chao1, Shannon, and Simpson) were significantly greater in high-CAP pepper varieties compared to their low-CAP counterparts (Figure 1C). This suggests that the high-CAP varieties harbor a more diverse and richer endophytic bacterial community. Furthermore, both PCoA and ANOSIM consistently revealed a significant influence of pepper variety on the composition of the endophytic bacterial community (Figure 1B). Therefore, varietal differences are a key factor influencing the diversity of endophytic bacteria in peppers. This result is consistent with previous findings in apple trees and Asparagus plants [46,47].

*Proteobacteria*, *Firmicutes*, *Bacteroidota*, and *Actinobacteriota* were the dominant phyla of endophytic bacteria in peppers (Figure 2), which aligns with previous research on rice and wheat [48,49]. *Proteobacteria*, *Actinobacteria*, and *Firmicutes* are widely distributed in nature and the soil [50]. They are also common in many plant tissues, playing a crucial role in maintaining the stability of the endophytic microbiota and participating in host metabolism [51]. Thus, it can be concluded that these microorganisms possess strong adaptability and can stably exist in plants and the soil. The composition of the endophytic bacterial community varies among different pepper varieties (Figure 2). Mamphogoro et al. [52] examined the bacterial communities on the surfaces of various *Capsicum annuum* fruits. Their results indicated that the pepper fruit surfaces were predominantly inhabited by the bacterial phyla *Proteobacteria*, *Firmicutes*, *Actinobacteria*, and *Bacteroidetes.* Our study revealed that the abundances of *Firmicutes* and *Bacteroidota* were significantly higher in YMH80 and HP03 than in TJ18 and TJ43 (Figure 3A,B). Intriguingly, YMH80 and HP03 also had significantly higher CAP levels than TJ18 and TJ43 (Supplementary Figure S1). Chen et al. [53] analyzed the gut microbiota of mice and found that capsaicin reduced the *Firmicutes*/*Bacteroidetes* ratio, beneficially reconstructing the microbial community and effectively alleviating obesity in mice. Although this phenomenon has not been observed in plants, we speculate that differences in the endophytic bacterial communities among various pepper varieties may be partially attributed to CAP content.

Furthermore, our study found that CAP content was positively correlated with *Enterobacter* and *Bacteroides* (Figure 4). High-CAP varieties exhibited significantly greater abundance of these genera compared to low-CAP varieties (Figure 3A,B). Inoculation with the endophytic bacterium *Enterobacter* significantly increased the CAP levels in peppers (Figure 6). Enterobacter strains are well-known nitrogen fixers [54]. Some nitrogen-fixing genera within the Enterobacteriaceae family have been isolated from various crops, such as rice, beans, and sugarcane, and have been shown to enhance nitrogen-fixing capabilities in these plants [55,56]. *Bacteroides*, belonging to the *phylum Bacteroidetes*, contribute to nitrogen acquisition [57]. Conversely, CAP content was negatively correlated with *Chujaibacter* and *Brochothrix* (Figure 4), and the relative abundances of these two genera were significantly lower in the high-CAP varieties compared to the low-CAP varieties (Figure 3A,B). It has been reported that *Chujaibacter* is a dominant population involved in nitrogen removal [58]. Moreover, the bacterial genera significantly correlated with CAP content were also the primary biomarkers identified via LEfSe analysis (Figure 3C). *Enterobacter* and *Bacteroides,* biomarkers of the high-CAP varieties (YMH80 and HP03), showed a positive correlation with CAP. Conversely, *Chujaibacter*, a biomarker of the low-CAP varieties (TJ18 and TJ43), exhibited a negative correlation with CAP (Figure 3C and Figure 4). Capsaicinoid biosynthesis itself is characterized by the convergence of the phenylpropanoid and branched-chain fatty acid pathways, a process that utilizes phenylalanine, valine, and leucine as three key nitrogen-containing precursors [33,59]. Multiple studies have reported a correlation between capsaicin content and available nitrogen content in pepper fruits [60]. Zhang et al. [61,62] showed that an appropriate amount of ammonium nitrogen, such as 25%, can promote the growth of sweet pepper plants. However, when the proportion of ammonium nitrogen reaches 50%, there is no significant change in capsaicin content. Collos et al. [63] showed that excessive levels of ammonium nitrogen are not conducive to optimal pepper growth and may even be toxic to the plants. These observations imply that appropriate levels of ammonium nitrogen can enhance capsaicin biosynthesis. Furthermore, these findings suggest that the endophytic bacterial community may regulate capsaicin biosynthesis by effectively utilizing available nitrogen in plants, as supported by our functional annotation analysis of the bacterial community. Notably, in high-CAP varieties (YMH80 and HP03), the abundances of bacterial OTUs associated with nitrogen fixation increased significantly, while those related to nitrate denitrification, nitrite denitrification, nitrite respiration, and nitrous oxide denitrification decreased markedly (Figure 5).

In summary, this study investigated the impact of various pepper varieties on the endophytic bacterial community and examined the role of these bacteria in enhancing the capsaicinoid (CAP) content of peppers. The findings reveal that the structure and composition of the endophytic bacterial community are significantly influenced by the pepper variety. Changes in the endophytic bacterial community are significantly associated with CAP levels in different pepper varieties. Moreover, we identified a beneficial *bacterium, Enterobacter*, which promotes capsaicin synthesis in peppers. These findings deepen our understanding of the intricate interactions between host plants and their microbiomes and suggest that manipulating plant endophytic microorganisms could be an effective strategy to increase CAP content in peppers. This study provides preliminary insights into the potential role of endophytic microbes in the biosynthesis of capsaicin in peppers. However, further investigations are needed to explore the molecular regulatory mechanisms by which endophytic microbial genes, metabolites, or proteins may influence capsaicin biosynthesis. Future studies will aim to elucidate the regulatory network involving endophytic microbes, plant varieties, and traits.

## Data Availability

The original contributions presented in this study are included in the article/Supplementary Material. Further inquiries can be directed to the corresponding author.

## Supplementary Materials

The following supporting information can be downloaded at https://www.mdpi.com/article/10.3390/microorganisms13030596/s1, Table S1. The capsaicin content of 100 pepper varieties. Figure S1. The capsaicin content of different pepper varieties. The error bars indicate the standard deviation (* *p* < 0.05, ** *p* < 0.01, *** *p* < 0.001, Student’s *t*-test). Figure S2. Rarefaction curve and rank abundance curve of OTUs in the four pepper samples. Figure S3: Taxonomic assignments and percent of community abundance at the phylum level in the endophytic bacteria of different pepper varieties. (A) Comparison of phyla exhibiting significant differences among different pepper varieties. The data were visualized by using STAMP (error bars represent Welch’s t-interval, * *p* < 0.05, ** *p* < 0.01, *** *p* < 0.001); (B) heatmap showing the abundance profile of dominant bacteria (top 10 phyla); (C) bacterial phylum biomarkers in pepper endophytes using LefSE analysis. The Kruskal–Wallis rank sum test was used to identify significantly different species within groups at an alpha of 0.05 and a threshold of 3.5.
