# Supplementary material for: The Changes of the Endophytic Bacterial Community from Pepper Varieties with Different Capsaicinoids
Source: Microorganisms. 2025 Mar 5;13(3):596. doi: 10.3390/microorganisms13030596 (PMC11946074; doi:10.3390/microorganisms13030596)
Supplement: Supplementary file 1 [file microorganisms-13-00596-s001.zip › microorganisms-3501949-supplementary.pdf]

Supplementary Materials

Table S1. The capsaicin content of 100 pepper varieties.

Numbering of *C. annuum*	Capsaicin Content (mg/g)
TJ18	0.80±0.01
TJ43	0.90±0.02
YJ26	2.00±0.37
YMH47	2.00±0.48
CXJ48	2.00±0.72
CT08	2.00±0.56
CS13	2.10±0.65
QT15	2.10±0.34
TJ39	2.10±0.16
CXJ37	2.14±0.51
CXJ51	2.20±0.73
TJ32	2.20±0.27
NJ31	2.90±0.63
NJ44	2.96±0.34
CXJ69	3.00±0.21
NJ38	3.20±0.32
CXJ49	3.30±0.34
NJ47	3.30±0.68
XJ24	3.40±0.50
CS12	3.50±0.53
CXJ62	3.70±0.74
HP05	3.70±0.66
YJ09	3.99±1.00
CS04	4.00±0.81
NJ07	4.50±1.01
CT34	4.70±0.78
YJ34	4.70±0.42
YJ38	4.70±0.67
YJ46	4.80±0.54
HL10	5.00±0.82
HP09	5.10±0.75
XJ22	5.10±1.07
CXJ56	5.10±0.59
XJ07	5.20±0.60
NJ37	5.40±0.20
YJ52	5.50±0.88
HP14	5.60±0.26
CT17	5.70±0.90

XJ25	5.70±0.64
YMH51	5.70±0.75
HP17	6.10±0.97
XJ12	6.10±0.35
XJ26	6.10±0.83
YJ33	6.20±0.72
YJ40	6.20±1.11
NJ39	6.20±0.98
CXJ12	6.60±0.62
NJ05	6.90±0.71
QT16	7.00±0.49
HL20	7.00±1.03
NJ35	7.00±0.89
NJ49	7.10±1.16
YJ18	7.20±1.12
YJ55	7.30±0.39
NJ50	7.60±1.97
NJ32	7.80±2.62
NJ28	8.00±0.68
YJ50	8.10±1.40
CS23	8.20±2.05
HL24	8.40±0.88
CXJ66	8.50±0.83
QT10	9.10±1.82
XJ11	9.60±2.32
NJ03	10.20±1.86
XJ08	10.30±1.25
CXJ27	11.00±2.08
HL03	11.20±2.20
HL27	12.10±0.80
YJ31	12.90±0.15
XJ19	13.10±0.76
NJ33	13.20±1.95
CS14	13.74±1.62
YJ42	14.20±0.82
CT32	14.60±2.53
NJ46	14.68±0.14
CT19	15.00±0.41
XJ30	15.10±3.09
YJ27	15.10±3.05
TJ25	15.20±2.23

CXJ64	15.50±2.43
HL23	15.70±1.68
YJ25	16.10±2.97
CT27	16.40±3.12
TJ13	17.17±1.81
TJ19	17.67±0.60
CXJ23	17.70±2.27
XJ20	17.80±2.47
HL08	18.00±0.90
CXJ80	18.40±1.92
CS20	20.10±2.68
YJ53	20.70±2.76
NJ29	22.70±1.70
CT28	23.70±1.19
XJ05	24.20±1.47
TJ15	24.80±1.39
TJ14	24.88±2.11
HL13	26.50±4.94
CXJ52	28.60±3.41
HP03	24.82±0.36
YMH80	35.00±1.57

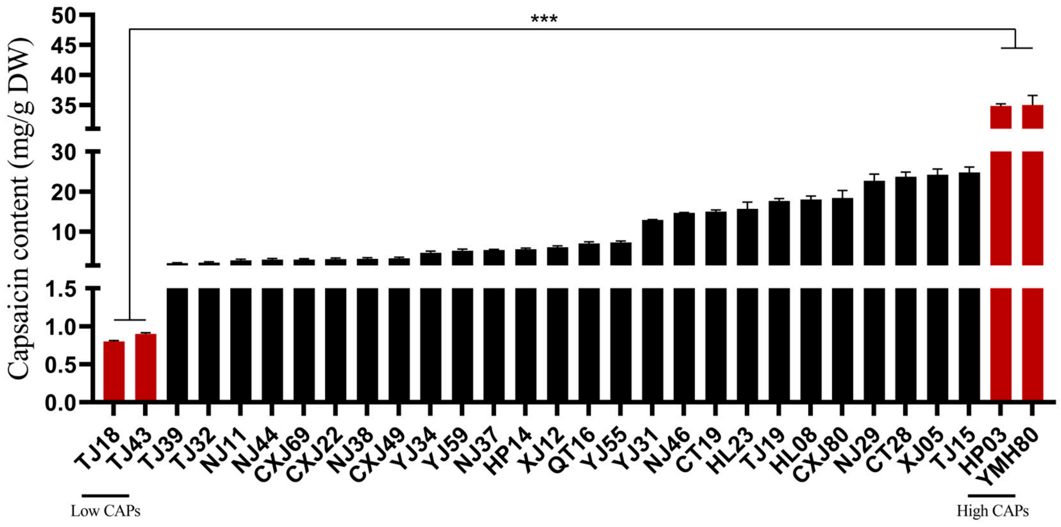

Figure S1. The capsaicin content of different pepper varieties. The error bars indicate the standard deviation (* $P < 0.05$, ** $P < 0.01$, *** $P < 0.001$, Student's *t*-test).

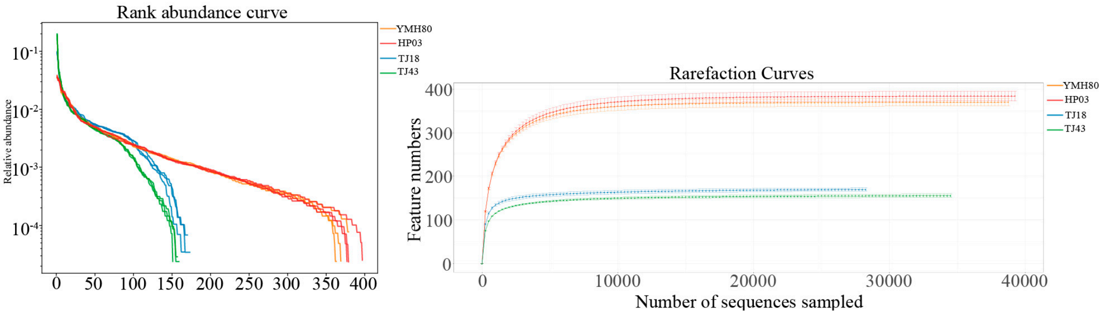

Figure S2. Rarefaction curve and rank abundance curve of OTUs in the four pepper samples.

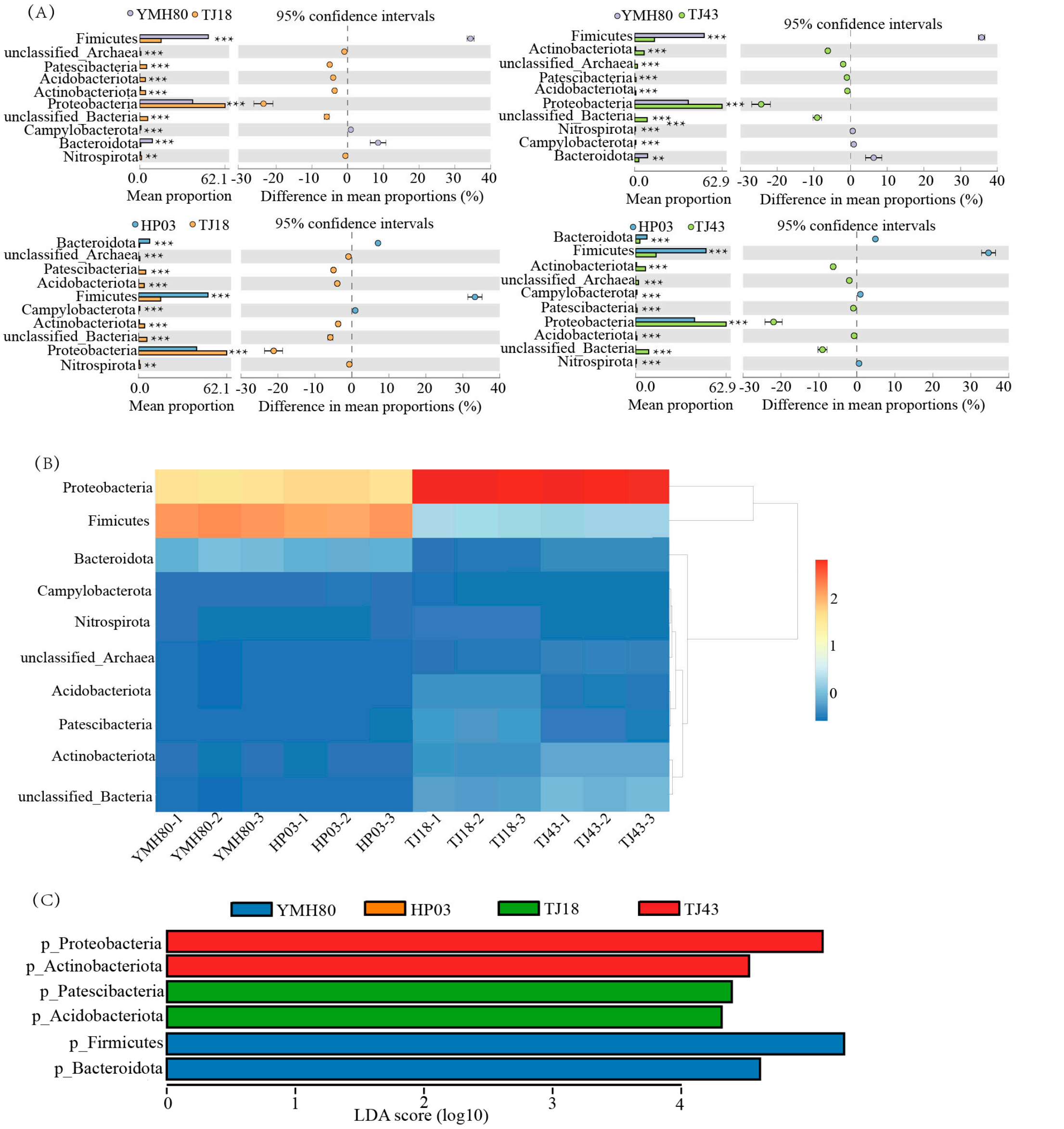

Figure S3. Taxonomic assignments and percent of community abundance at the phylum level in the endophytic bacteria of different pepper varieties. (A) Comparison of phyla exhibiting significant differences among different pepper varieties. The data were visualized by using STAMP (error bars represent Welch's t-interval, * $P < 0.05$, ** $P < 0.01$, *** $P < 0.001$); (B) Heatmap showing the abundance profile of dominant bacteria (top 10 phyla); (C) Bacterial phylum biomarkers in pepper endophytes using LefSE analysis. The Kruskal–Wallis rank sum test was used to identify significantly

different species within groups at an alpha of 0.05 and a threshold of 3.5.
